# Surgical Notes from the *Lancet*

**Published:** 1861-12

**Authors:** 


### SURGICAL NOTES FROM *THE LANCET.*

The following essentially practical observations, we condense from a paper in the December number of the London *Lancet*, by Frederic C. Skey, Esq., F. R. S., Surgeon to St. Batholomew's Hospital. They are in brief, the general principles as carried out in Mr. Skey's wards in that institution, and will repay perusal:

*Fractures of the Leg.*—A well-made flock pillow, long enough to extend above the knee, and some inches below the foot, is an efficient, and most comfortable agent in the treatment of simple fractures of the leg, where the displacement is not great, nor the fracture very oblique.

When bound on to the leg by means of four or five straps with buckles, which are drawn very tight, the pressure, as is known, is diffused over the entire surface of the leg, and is unproductive of pain, or of such an amount of discomfort as to prevent sleep.

*Strangulated Hernia.*—One word on the subject of the division of the sac in cases of hernia. The operation of non-division has, I confess, disappointed me; and I am disposed to concur with many other authorities in the belief that the advocates of the operation, are not borne out in their estimate of its supposed advantages—such, at least, is the result of my own personal, and necessarily limited experience in the practice of St. Bartholomew's Hospital. The operation for strangulated hernia, although the theory is obvious enough, and the object to be obtained, clearly understood, is often an operation of considerable difficulty and of anxiety to the surgeon operating; and in a large number of cases, a young surgeon is not indisposed to suspend for the moment, his "pride of place," and to avail himself of the advice and co-operation even of his assistant, certainly of his seniors, should such be present. I consider the difficulty of the operation, as a rule, considerably increased by the attempt to divide the stricture from without inwards, or, in other words, without opening the sac which surrounds the intestine.

*Lithotrity.*—In Skey's opinion, there are comparatively few cases of calculus, in which the operation of lithotrity is not available. He has adopted a rule never to operate with the lithotrite, unless the urethra will admit a catheter of the size of No. 9 or 10. Among others, he cites one case of a man, aged seventy-two, upon whom he operated with this instrument, no less than seventeen times. The stone was of lithic acid, and his experience shows this composition, and not oxalate of lime, forms the hardest calculi.

*Stimulating Treatment of Burns.*—In reference to the subject of burns, I have always been an advocate for the stimulating principle of treatment first suggested by Dr. Kentish. That principle, to my observation apparently so sound, has been repudiated of late years, by many eminent surgeons. The thoroughly negative action of carron oil and lime-water has been substituted for it, from which, I confess, I have never seen a corresponding benefit obtained, It is notorious, that the intense pain caused by a drop of hot sealing-wax, or other similar agent, on the skin, is much mitigated by the temporary application of great heat. The pain subsides in the course of a few moments, by holding the affected part to the fire. The explanation is not, perhaps, very obvious, but the fact remains.

In July last, five men were admitted into St. Bartholomew's Hospital, with severe burns in the face, head, chest, and arms. One died immediately after his arrival. My house-surgeon, Mr. Richard Smith, was at the time ill, and the immediate charge of these cases devolved on the colleague, who applied the oil and lime-water to the arms and hands of each of the surviving men. At that moment Mr. Smith arrived, and completed the dressing by the application of a solution of nitrate of silver, (ten or twelve grains to the ounce of water) to the face, neck, etc. I did not see these men till the following morning, when all four complained of severe burning pain in the arms and hands, but stated they were free from pain in the other affected parts. The stimulating solution above mentioned, was applied to the upper extremities, and the relief at the expiration of about a quarter of an hour was complete. In two of the cases, some pain returned on the third day, and relief was obtained by the same means. My directions are simply these: In the case of infants or young children, wash the affected surface, if not very extensive, with a solution of nitrate of silver, in strength, six or eight grains to the ounce, and immediately cover up the part with a thick mass of cotton-wool; in the case of an adult, from twelve to fifteen grains, unless the surface requiring the application be very large. Should pain return, the solution may be advantageously resorted to at any early stage of the treatment.

*Housemaid's Bursæ.*—Placing no reliance on blisters and iodine, he reduces the bursa to an abscess, by passing a full sized thread through the centre of the swelling; this thread is removed, in from two to five or six days, during which interval suppuration will inevitably ensue, and the case is then treated like any other, of abscess. Between 100 and 200 cures effected in this way attest its merit.

The same agent, and the same principle, is equally applicable to *ranula.* Indeed, it is quite remarkable with what rapidity this disease recedes under the action of the thread, whether the cyst be of average or of the largest size.

*Wounds into Joints.*—We only allude to this to express surprise at what seems Mr Skey's want of familiarity with Barwell's recent excellent work on the Joints. Mr. S. mentions the case of a boy, admitted with a small wound on the inner side of the knee-joint, from which synovia escaped after his admission, and subsequently extensive suppuration occurred. He queries, after detailing the case, "Can this boy recover the former mobility of the joint, or is anchylosis inevitable?" He believes the functions of the joint may be restored; but "as far as he knows," "the subject (the result of suppurative synovitis), has not been investigated," though "not devoid of surgical interest." Barwell would seem to have pretty clearly settled, that anchylosis is by no means inevitable in these cases,—*vide* his *Diseases of the Joints*, chap. I., p. 74, *et seq.*, American edition.

*Hysteria.*—In concluding his paper, he desires to "direct the especial attention of the younger members of the surgical profession to one of the most remarkable features associated with the treatment of disease. I allude to the great prevalence of *hysteria* in the cases of young women. It is quite remarkable how general is its presence, and how frequently it is interwoven with the symptoms of real disease. Abundance of cases recur to my recollection during the past year; reputed affections of the spine, and of the knee-joint more especially, at first sight of the most deceptive character. I would go so far as to assert. that its presence, in some degree or other, is almost universal. And yet how critically important is a correct diagnosis in such examples! What form of treatment has a greater tendency to aggravate the symptoms of hysteria, than that which may be judiciously applied to the cure of real diseases?

